# Axonal and glial microstructural information obtained with diffusion-weighted magnetic resonance spectroscopy at 7T

**DOI:** 10.3389/fnint.2013.00013

**Published:** 2013-03-13

**Authors:** Itamar Ronen, Ece Ercan, Andrew Webb

**Affiliations:** C.J. Gorter Center for High Field MRI, Department of Radiology, Leiden University Medical CenterLeiden, Netherlands

**Keywords:** tissue microstructure, magnetic resonance spectroscopy, high field MRI, diffusion-weighted imaging, white matter, N-acetylaspartate, choline, creatine

## Abstract

Diffusion-weighted magnetic resonance spectroscopy (DWS) offers unique access to compartment-specific microstructural information on tissue, and potentially sensitive detection of compartment-specific changes in disease. The specificity of DWS is, however, offset by its relative low sensitivity, intrinsic to all MRS-based methods, and further exacerbated by the signal loss due to the diffusion weighting and long echo times. In this work we first provide an experimental example for the type of compartment-specific information that can be obtained with DWS from a small volume of interest (VOI) in brain white matter. We then propose and discuss a strategy for the analysis of DWS data, which includes the use of models of diffusion in compartments with simple geometries. We conclude with a broader discussion of the potential role of DWS in the characterization of tissue microstructure and the complementarity of DWS with less-specific but more sensitive microstructural tools such as diffusion tensor imaging.

## Introduction

The characteristic dimensions of microstructural features of living tissue are several orders of magnitude smaller than the typical spatial resolution obtained by magnetic resonance imaging (MRI). Yet, MRI has become a ubiquitous tool for investigating tissue microstructure *in vivo*, thanks to the direct relationship between the motional properties of the nuclei that generate the MR signal and the properties of the NMR signal that ensues. In particular, self-diffusion of spins in a magnetic field gradient generates a signal loss that is controlled by the characteristics of the gradients as well as by geometric factors that hinder or restrict the diffusion process over time. Incorporation of pulsed magnetic field gradients in MR pulse sequences led to the most robust manner of sensitizing the MR signal to self-diffusion (Stejskal and Tanner, 1965) and has since been frequently used in the investigation of numerous aspects of tissue microstructure, including characterization of disease processes (Le Bihan et al., 2001), exploration of white matter connectivity (Conturo et al., 1999; Melhem et al., 2002), and quantitative evaluation of microstructural features such as axonal diameter and myelination (Nomura et al., 1994; Neil et al., 1998; Assaf et al., 2008).

The explanatory power of diffusion-weighted (DW) MRI methods is limited, however, by the lack of specificity of the signal source, namely, the hydrogen protons in water molecules. The ubiquity of water in living tissue (~65% in volume) accounts for the high sensitivity of MRI when compared to MR of other tissue constituents, but the lack of compartmental-specificity complicates the interpretation of DW-MRI results. For example, competing disease processes that simultaneously act on several tissue compartments may have similar, or opposed effects on diffusion properties of water in those compartments. This, in turn, generates a high degree of ambiguity in the interpretation of the DW-MRI results, or in other instances dilutes the information related to the specific process of interest. Examples include DW-MRI of stroke, where the decrease in apparent diffusion coefficient (ADC) in the ischemic region can be attributed to several competing effects that influence the ADC: increase in restriction in the diffusion of water in the extracellular compartment, influx of water into the restricted intracellular compartment, and the cessation of active transport (cytosolic flux) in the affected cells. In the case of multiple sclerosis (MS) and other neurological disorders, damage to myelin may increase water ADC and reduce the fractional anisotropy (FA) of the diffusion tensor, but neuronal/axonal damage may reduce overall ADC. It is thus desirable to devise a MR-based modality with an intrinsic ability to provide compartment-specific information about tissue microstructure. Several approaches to achieve this goal have been suggested, including high-level modeling of DW-MRI acquired at a variety of diffusion-weighting values and directions (Assaf and Basser, 2005; Zhang et al., 2012). A more direct way to tackle the issue of compartment-specificity is to measure the diffusion properties of endogenous spin species that reside in specific tissue compartments and are concentrated enough to yield a visible MR signal with reasonable signal-to-noise ratio (SNR). In neural tissue, these are intracellular metabolites in the mM concentration range and give rise to distinct spectroscopic patterns in a magnetic resonance spectroscopy (MRS) experiment. Some metabolites are almost entirely cell-specific, e.g., N-acetylaspartate (NAA) and the excitatory neurotransmitter glutamate (Glu), which reside both almost exclusively in neurons/axons, and myo-inositol (mI) and glutamine (Gln), which are thought to primarily reside in astrocytes (Brand et al., 1993; Choi et al., 2007). Other intracellular metabolites, e.g., creatine and phosphocreatine (Cr and PCr) are less-specific and have a well-defined role in cellular metabolism, while others are preferential to a specific cell type but not exclusively so, e.g., choline-based molecules, which are believed to be more concentrated in glia than in neurons at a ratio of approximately 3:1 (Choi et al., 2007). Given the low concentration of these metabolites, the sensitivity of their detection is much lower than that of water, and thus the spatial resolution that can be achieved with diffusion-weighted spectroscopic measurements is similar to that of MRS methods in general, i.e., typical size of a volume of interest (VOI) in human experiments is several milliliters.

Diffusion-weighted MRS (DWS) studies have been used to probe excised neural tissue (Assaf and Cohen, 1998a,b); *In vivo* DWS studies in small animals and primates were performed on muscle (Moonen et al., 1990; van Doorn et al., 1996; de Graaf et al., 2000) and brain (Merboldt et al., 1993; van der Toorn et al., 1996; Pfeuffer et al., 2000; Dreher et al., 2001; Valette et al., 2007; Marchadour et al., 2012). Robust measurements have been performed in humans, (Kroenke et al., 2004; Ellegood et al., 2005a,b; Upadhyay et al., 2007, 2008). DWS studies at 7T provided tissue-specific information on several metabolites (Kan et al., 2012) and provided sensitive diffusion tensor MRS (DTS) data on NAA from small portion of the corpus callosum in a group of subjects with MS (Wood et al., 2012).

In this work we explore the potential of DWS to provide compartment-specific information by focusing on a set of DWS results from a small portion of the corpus callosum. We show that the DWS data *per se* dependably reflects microstructural features of the structures populated by the metabolites on which the measurements are performed. We then discuss the potential role that DWS may have in the evaluation of microstructural properties of tissue in conjunction with other methods, and its optimal positioning in the arsenal of MR-based microstructural tools.

## Materials and methods

### Human subjects

A total of 12 healthy volunteers (6 females, 6 males, age: 26 ± 4 years) participated in this study. The study adhered to the LUMC Institutional Review Board guidelines and informed consent was obtained from all subjects prior to the study.

### MRI scanner/hardware

All experiments were performed on a 7 Tesla Philips Achieva whole-body MRI scanner (Philips Healthcare, Best, The Netherlands) equipped with gradient coils capable of a maximum gradient strength of 40 mT/m and a slew rate of 200 T/m/s. A head coil consisting of a quadrature birdcage transmit and 32-channel phased array receive was used for all measurements (Nova Medical Inc., Wilmington, MA, USA).

### MRI/MRS protocols

#### Anatomical images

A short survey scan and a sensitivity encoding (SENSE) reference scan were followed by a 3D T_1_-weighted gradient echo acquisition to allow for accurate planning of the experiment. Imaging parameters were: field of view (AP, FH, RL): 246.4 × 246.4 × 174.0 mm^3^, resolution 1 × 1 × 1 mm^3^, TR/TE = 4.7/2.1 ms. Total scan time—3 min.

#### DTI protocol

A single subject (male, 25 years) was scanned. Multi-slice single-shot 2D spin-echo echo planar imaging (EPI) (60 slices of 2 mm thickness, TR/TE = 9100 ms/58 ms), field of view of 224 × 224 × 120 mm^3^ with an in-plane data matrix of 112 × 112, resulting in a data set with resolution of 2 × 2 × 2 mm^3^, 32 diffusion-weighting directions with *b* = 1000 s/mm^2^. Fat suppression was performed using spectral inversion recovery (SPIR). Parallel imaging was performed using SENSE with a reduction factor of 3 along the phase encoding direction (AP). Total scan time—6 min.

#### DWS

**Figure 1:**
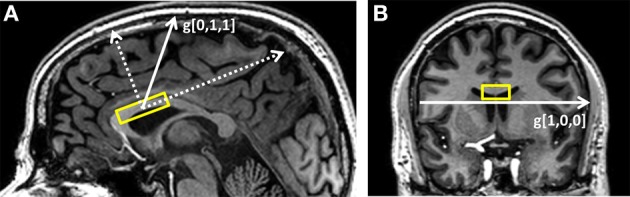
**Typical placement of the DWS VOI (A) on the sagittal plane and (B) on the coronal plane.** The gradient directions used in the experiment are *g*[0,1,1], which is mostly perpendicular to the callosal fibers, and *g*[1,0,0], which is mostly parallel to them.

The VOI for the experiment was the anterior body of the corpus callosum (Witelson segments A3–A4 and part of A2, see Figure 1 for a typical VOI positioning). VOI dimensions were 25 mm(a–p), 10 mm(r–l), and 8 mm(f–h) for a total volume of 2 cc. Slight variations in positioning of the VOI across subjects were necessary in order to adjust to slightly different patterns of anterior-posterior curvature. The sequence used for the DWS experiment was a point-resolved spectroscopy (PRESS) sequence (TE = 121 ms) supplemented with a bipolar diffusion-weighting scheme (Kan et al., 2012). Cardiac synchronization on every 3rd cardiac cycle was achieved via a pulse-oximeter resulting in a TR of about 3 s and was used to avoid strong fluctuations in signal intensity due to cardiac pulsation. Number of time-domain points = 2048, spectral width = 3000 Hz. Shimming up to second order was performed with pencil beam excitation and a typical tNAA line width was about 10 Hz throughout the experiment.

Two directions with respect to the VOI coordinates were chosen for the diffusion weighting: (1) a pure right-left direction, thus mostly parallel to the direction of the callosal fibers); (2) one mostly perpendicular to the callosal fibers, forming a 45° angle between the anterior-posterior axis and the inferior-superior axis of the VOI (see diagram in Figure 1). These gradient directions can be denoted in the VOI coordinates as [1,0,0] and [0,1,1], and $g_{\left[0,1,1\right]}=\sqrt{2}\cdot g_{\left[1,0,0\right]}$ for a specific choice of gradient strength. Diffusion-weighting parameters were: gradient pulse duration: 34 ms, diffusion time (Δ): 60.5 ms, 8 equally spaced gradient amplitude values between 0 and 3.15 gauss/cm resulting in a set of *b*-values of 0–6594 s/mm^2^ for *g*_[0,1,1]_ and 0–3297 s/mm^2^ for *g*_[1,0,0]_. Each of the 15 direction/gradient amplitude conditions (2 directions × 7 gradient values + one acquisition without diffusion weighting) was acquired 48 times for a total of set of 720 individual free induction decays (FIDs) and a total scan time of ~35 min. “Weak” water suppression was performed in order to preserve enough of the water signal (about 5–10 times the NAA singlet for all conditions) for subsequent phasing and frequency drift correction for each individual acquisition. To help enable this condition for the higher *b*-values, water suppression was performed in a suboptimal manner by providing only a fraction of the RF power needed for full water suppression. Following the DWS experiment, a short DWS acquisition (~3 min) with the same diffusion-weighting conditions and four acquisitions/condition was performed without water suppression and subsequently used for eddy current correction.

Given the relatively large chemical shift displacement caused by the relatively low bandwidth of the 180° pulses in the PRESS sequence (about 4 mm for the chemical shift difference between the tNAA singlet (2.0 ppm) and the choline singlet (3.2 ppm) in the FH and LR directions), six of the subjects (4 females, 2 males, 25.7 ± 4.8 years) were scanned with the frequency centered around the tNAA peak and the other six (2 females, 4 males, 27.3 ± 3.4 years) centered around the choline peak. For the six subjects scanned with the frequency at 2.0 ppm we report only the tNAA results, and for those scanned with the frequency at 3.2 ppm, results are reported for both tCho and tCr, as the displacement of about 0.5 mm between the choline and the Cr/PCr singlet was deemed negligible.

### Post-processing and analysis

**Figure 2:**
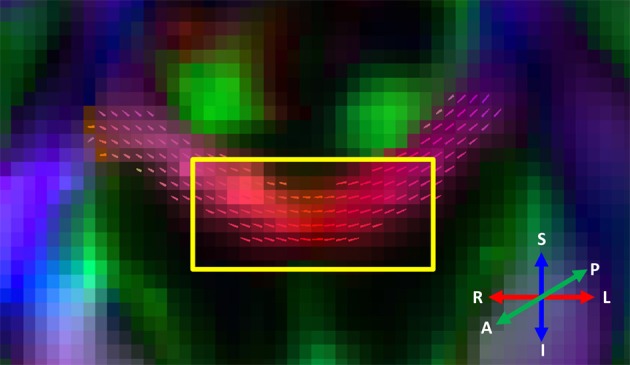
**Color-coded FA map of a coronal slice through the body of the corpus callosum.** The DWS VOI dimensions (20 mm RL × 8 mm FH) in that plane are represented by the yellow rectangle. The ticks in each voxel represent the direction of the main diffusion eigenvector. The resulting directional range is significant, approximately ±20°.

DTI processing was performed using ExploreDTI (Leemans et al., 2009). A color-coded FA map and a 2D main eigenvector tick map were generated and displayed in Figure 2.

### DWS data processing

Data processing was performed using a custom written program in Matlab® (Mathworks Inc, Natick, MA, USA) as described previously (Kan et al., 2012). The main steps in the data processing included: weighted summation and phasing of the individual outputs of the 32 receive coils based on reference water signal, eddy current correction, zero-order phase correction, frequency drift correction, and subsequent averaging. The resulting spectra were analyzed using LCModel (Provencher, 1993) with an appropriate simulated set of basis spectra.

### Modeling of the DWS diffusion data

Analysis of DWS data has been performed in two ways. First, a non-biased analysis was carried out, where general diffusion properties for the three main metabolites were calculated: *D*_‖_, *D*_⊥_, ADC, and pseudo-FA (*D*_‖_/*D*_⊥_). These properties were calculated based on the assumption of monoexponential decay with respect to *b*-value:

(1) $$S(b_{i})=S(\text{0})\;\cdot \;e^{-b_{i}\cdot D_{i}}$$

where *S*(*b*) is the measured signal, *S*(0) is the signal without diffusion weighting, *b*_*i*_ is the *b*-value in the direction *i* and *D* is the calculated diffusion coefficient in that direction.

### Modeled analysis of the DWS signal

Expressions for signal attenuation of the NMR signal in the presence of magnetic field gradients, when the spin motion is restricted was first formulated by Neuman (1974) and applied to the Stejskal-Tanner experiment by Murday and Cotts (1968). The theory was extended to cover a variety of geometries (Balinov et al., 1994; van Gelderen et al., 1994) and succinctly formulated in the following expression (Aslund and Topgaard, 2009) for planar, cylindrical, and spherical pores:

(2) $$\ln\left(S_{⊥}\right)=-2\gamma^{2}{\vec{g}}_{⊥}^{2}\sum_{m=1}^{\infty }\frac{1}{\alpha_{m}^{2}\left(\alpha_{m}^{2}R^{2}-n_{D}\right)}\times \frac{2\alpha_{m}^{2}D\delta -2+2e^{-\alpha_{m}^{2}D\delta }+2e^{-\alpha_{m}^{2}D\Delta }-e^{-\alpha_{m}^{2}D\left(\Delta -\delta \right)}-e^{-\alpha_{m}^{2}D\left(\Delta +\delta \right)}}{{\left(\alpha_{m}^{2}D\right)}^{2}}$$

Where *n*_*D*_ = 2 for cylinders and 3 for spheres. α_*m*_ satisfies the equation *j*′_1_(α_*m*_*R*) = 0, where *j*′_1_ is the derivative of the Bessel function (for cylinders) or spherical Bessel function (for spheres) of the first kind and first order, and *R* is the radius of the cylinder or sphere, respectively.

### Modeling the tNAA DWS signal

Analysis of the tNAA signal was based on the assumption that in white matter tNAA resides exclusively in axons. An estimate of the typical axonal diameter in the callosal region in which the DWS measurements were performed was obtained from (Aboitiz et al., 1992). Accordingly, about 75% of axons in the region of interest possess an inner diameter *D*_ax_ such that *D*_ax_ < 1.5 μm, and for 24% of these axons 1.5 μm < *D*_ax_ < 4.5 μm. These values take into account about 30% post-mortem shrinkage in the reported values, as described in Aboitiz et al. (1992). Axonal geometry inside the VOI is assumed to be a stack of curved cylinders, possessing a macroscopic curvature as well as a microscopic misalignment, or “fanning.” In DTI analysis, macroscopic curvature of axonal tracts can be for the most part neglected, but in the case of DWS the dimensions of the VOI are of the same order of magnitude of the radius of curvature of the tract and thus the curvature cannot be neglected. This can be seen in Figure 2, in which a coronal slice of a directionally color-coded FA map is shown, along with a representation of a typical VOI in that plane. The variability of the fiber directionality within the VOI, as represented by the projections of the main diffusion tensor eigenvectors on the plane, is clearly visible. Significant microscopic misalignment has been reported in the corpus callosum of the rat (Leergaard et al., 2010) and the macaque monkey (Sotiropoulos et al., 2012), and is reported in humans in this research topic by Budde et al., and thus has to be introduced as well.

We base our formulation of the diffusion of tNAA in the axons on previous works (Assaf et al., 2004; Avram et al., 2008). For a single axon at an angle ψ to the direction of the gradient, the diffusion-weighted signal *S* is the product of the diffusion perpendicular and parallel to the axonal wall:

(3) $$\begin{array}{l} S\left({\vec{q}}_{j},\;\Delta \right)=S_{⊥}\left({\vec{q}}_{j_{⊥}},\;\Delta \right)\;\cdot \;S_{\|}\left({\vec{q}}_{j_{\|}},\;\Delta \right) \end{array}$$

where Δ is the diffusion time, ${\vec{q}}_{\text{​}j}=\frac{\delta \gamma {\vec{g}}_{\text{​}j}}{2\pi }$ (δ is the duration of the pulsed field gradient, γ is the gyromagnetic ratio of protons, ${\vec{g}}_{\text{​}j}$ is the diffusion-weighting gradient vector in the direction *j*), ${\vec{q}}_{j\|}={\vec{q}}_{j}\;\cdot \;\cos\;\psi_{ij}\;$ and ${\vec{q}}_{j⊥}={\vec{q}}_{j}\;\cdot \;\sin\;\psi_{ij},\;\pi /2-\psi_{ij}$ is the angle between ${\vec{q}}_{j}$ and an axon in direction *i*. *S*_⊥_ is given by Equation (1) expressed for the cylindrical case, and *S* is assumed to be governed by non-restricted diffusion, i.e.:

(4) $$\begin{array}{l} S_{\|}({\vec{b}}_{\|},\;\Delta )=S_{\|}(0)\;\cdot \;e^{-{\vec{b}}_{\text{​}\|}\cdot D} \end{array}$$

where ${\vec{b}}_{\|}=\gamma^{2}\delta^{2}{\vec{g}}_{\|}^{2}(\Delta -\frac{\delta }{3})$ and *D* is the diffusion coefficient of tNAA in the cytoplasm, from here onward D(tNAA).

To account for both the macroscopic curvature within the VOI and the microscopic axonal misalignment, a distribution of the angle ψ_*ij*_ is introduced, where ψ_*ij*_ = θ_*ij*_ + φ_*ij*_, where θ_*ij*_ is the macroscopic contribution and φ_*ij*_ accounts for axonal misalignment. It is assumed that the displacement of NAA molecules during the diffusion time Δ is much smaller than the radius of curvature, hence θ_*ij*_ along this distance remains constant. In this case expression (3) is modified to yield the following form (Avram et al., 2008):

(5) $$\begin{array}{l} S\left({\vec{q}}_{j},\;△\right)=\frac{\sum_{i=1}^{N}p\left(\psi_{ij}\right)\;\cdot \;\sin\left(\psi_{ij}\right)\;\cdot \;S_{⊥}\left(q_{j}\;\cdot \;\cos\left(\psi_{ij}\right),\;\Delta \right)\;\cdot \;S_{\|}\left(q_{j}\;\cdot \;\sin\left(\psi_{ij}\right),\;\Delta \right)}{\sum_{1}^{N}p\left(\psi_{ij}\right)\;\cdot \;\sin\left(\psi_{ij}\right)} \end{array}$$

For simplicity, it is assumed that the angles ψ_*ij*_ belong to a Gaussian distribution with zero mean and standard deviation σ_ψ_:

(6) $$p\left(\psi \text{|}0,\;\sigma_{\psi }\right)=\frac{\text{1}}{\sigma_{\psi }\sqrt{\text{2}\pi }}e^{\frac{\text{-}\psi^{\text{2}}}{\text{2}\sigma_{\psi }^{\text{2}}}}$$

**Figure 3:**
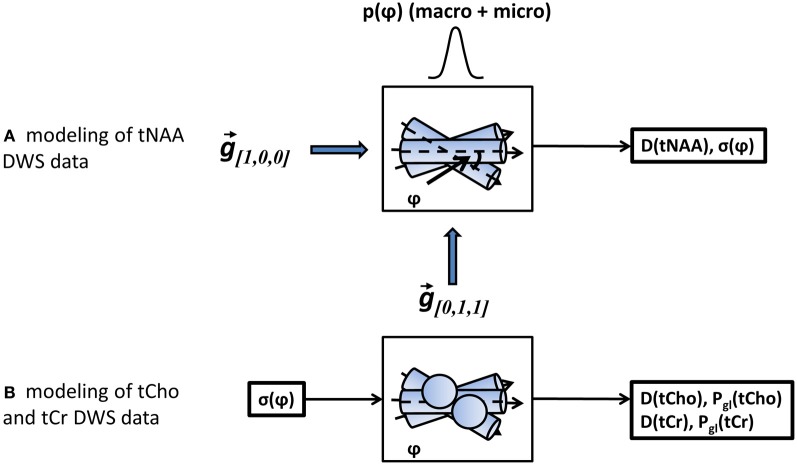
**A schematic representation of the modeling steps.** In step **(A)** the tNAA DWS data in both diffusion-weighting directions is fitted to a model of diffusion in a set of misaligned cylinders with a gaussian angular distribution with standard deviation σ_ψ_. The fitting variables are the diffusion coefficient of tNAA and σ_ψ_. In step **(B)**, the DWS signal of the two non-axonal specific metabolites, tCho and tCr is fitted to a mixed model of cylinders (axons) and spheres (glia), with σ_ψ_ obtained earlier as one of the input parameters. The fitting variables for each metabolite data are its diffusion coefficient, D(tCho) or D(tCr), and its glial fraction, *P*_gl_(tCho) or *P*_gl_(tCr), respectively.

A more appropriate description of angular distribution for the three-dimensional fanning of axons about a specific direction is the Bingham distribution (Bingham and Mardia, 1978), applied in a number of studies that examined axonal fanning based on diffusion-weighted images (Kaden et al., 2007; Sotiropoulos et al., 2012). Here we chose the Gaussian distribution for simplicity, keeping in mind that the actual angular distribution, and in particular the portion attributed to the macroscopic curvature of the axonal tract, will be best described empirically rather than analytically. As the last step, the diffusion-weighted data in both directions was simultaneously fitted to the model in Equation (5) using two free-fitting variables: D(tNAA), the diffusion coefficient of tNAA in the direction parallel to the fibers, and σ_ψ_, the standard deviation of the total axonal dispersion. A diagram of the analysis procedures is given in Figure 3.

### Modeling the tCr and tCho DWS signal

Analysis of the tCr and tCho signals must assume a significant contribution from glial content of these metabolites. The geometric representation of glia is rather complex, as the cells consist of a relatively large, oblong cell body with long, thin projections (Kuehnel, 2003; Verkhratsky and Butt, 2007). One extreme simplification is to assume that most of the cellular volume is represented by the cell body, approximated by a spherical pore. In order to obtain a representative compartment size, it is then possible to use Equation (2) for the spherical case. Since neither tCho nor tCr are exclusively astroglial, a complete analysis for *S*_*M*_, the diffusion-weighted signal for the metabolite in question, should include an axonal fraction and a glial fraction, *P*_*M*,_gl__ and *P*_*M*,_ax__, respectively, weighing the DWS signal contribution from these compartments, *S*_*M*,_gl__ and *S*_*M*,_ax__:

(7) $$S_{M}[\delta ,\;\Delta ,\;{\vec{g}}_{i}]=P_{M,\text{ gl}}\;\cdot \;S_{M,\text{ gl}}\left(r_{\text{gl}},\;D_{M},\;\left[\delta ,\;\Delta ,\;{\vec{g}}_{i}\right]\right)+P_{M,\text{ ax}}\;\cdot \;S_{M,\text{ ax}}(r_{\text{ax}},\;D_{M},\;\sigma_{\Psi },\;[\delta ,\;\Delta ,\;{\vec{g}}_{i}])$$

Here, *D*_*M*_ is the diffusion coefficient of the metabolite, *r*_gl_ is the typical radius of the glial cell body (assumed to be a sphere), *r*_ax_ is the typical axonal diameter and σ_ψ_ is the axonal angular dispersion. If σ_ψ_ is previously estimated through the DWS of the axonal constituent (tNAA), it can be used in fitting Equation 7 to the DWS data of the tCr or tCho, for further estimation of *P*_*M*,_gl__, *P*_*M*,_ax__, and *D*_*m*_.

### Statistical analysis of residuals for the modeled data

The quality of the fit of the experimental DWS data to the proposed models was assessed performing a χ^2^ goodness-of-fit test for the residuals. For this purpose, we used the Cramér-Rao Lower bounds (CRLB) of the spectroscopic measurements for the value of the variance of the single measurement. Typical CRLB values for the condition *b* = 0 s/mm^2^ (in percent of the estimated peak integral) were 4% for the tNAA, 6% for the tCr, and 6% for the tCho. The highest CRLB values were those for the maximum *b-value* in the [1,0,0] condition and typical values were 18% for the tNAA, 13% for the tCr, and 13% for the tCho.

## Results

**Figure 4:**
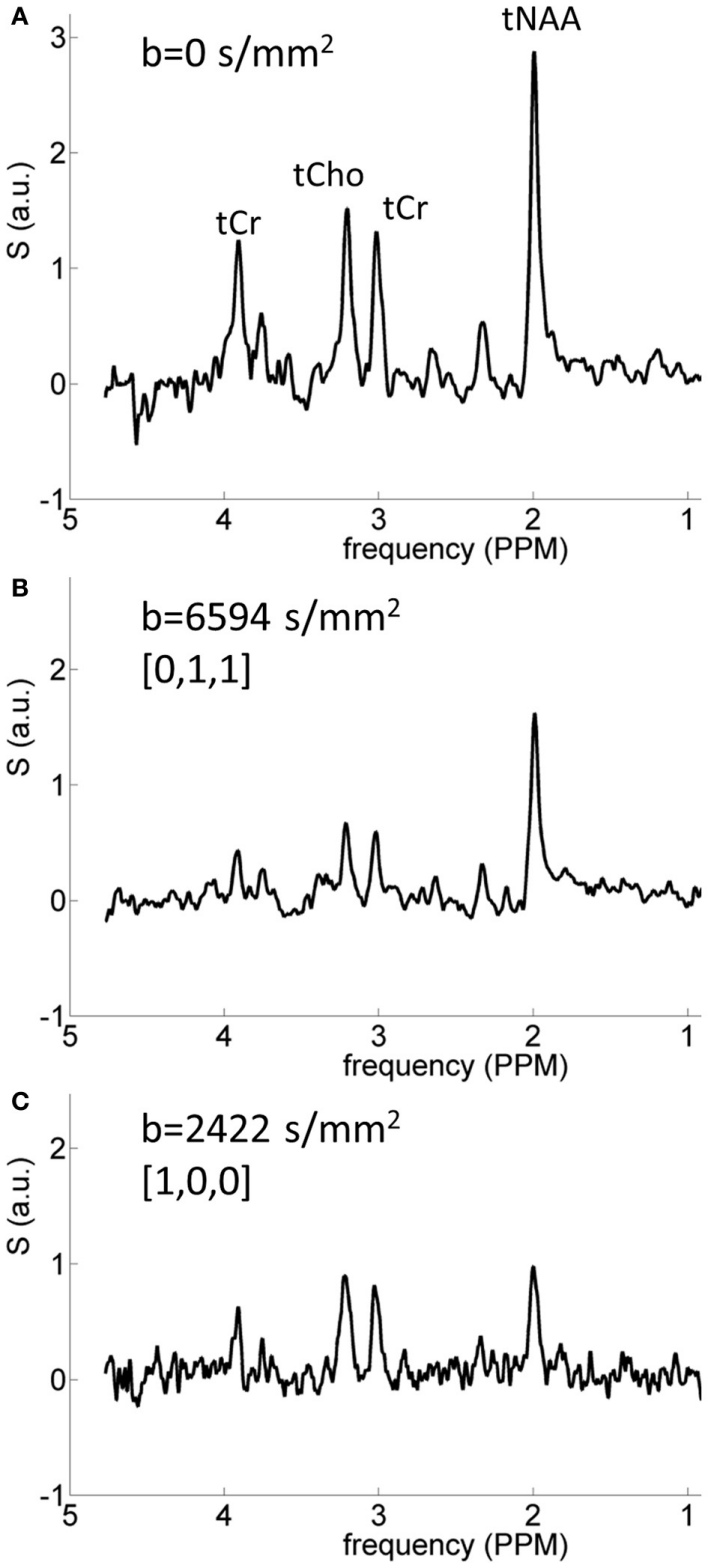
**Typical spectra acquired from one of the subjects. (A)** Spectrum acquired without diffusion weighting; **(B)** spectrum acquired in the direction [0,1,1] with *b* = 6594 s/mm^2^, the largest *b*-value used here; **(C)** spectrum acquired with *b* = 2422 s/mm^2^ in the direction [1,0,0]. The strong diffusional anisotropy of the tNAA signal is clearly seen.

**Figure 5:**
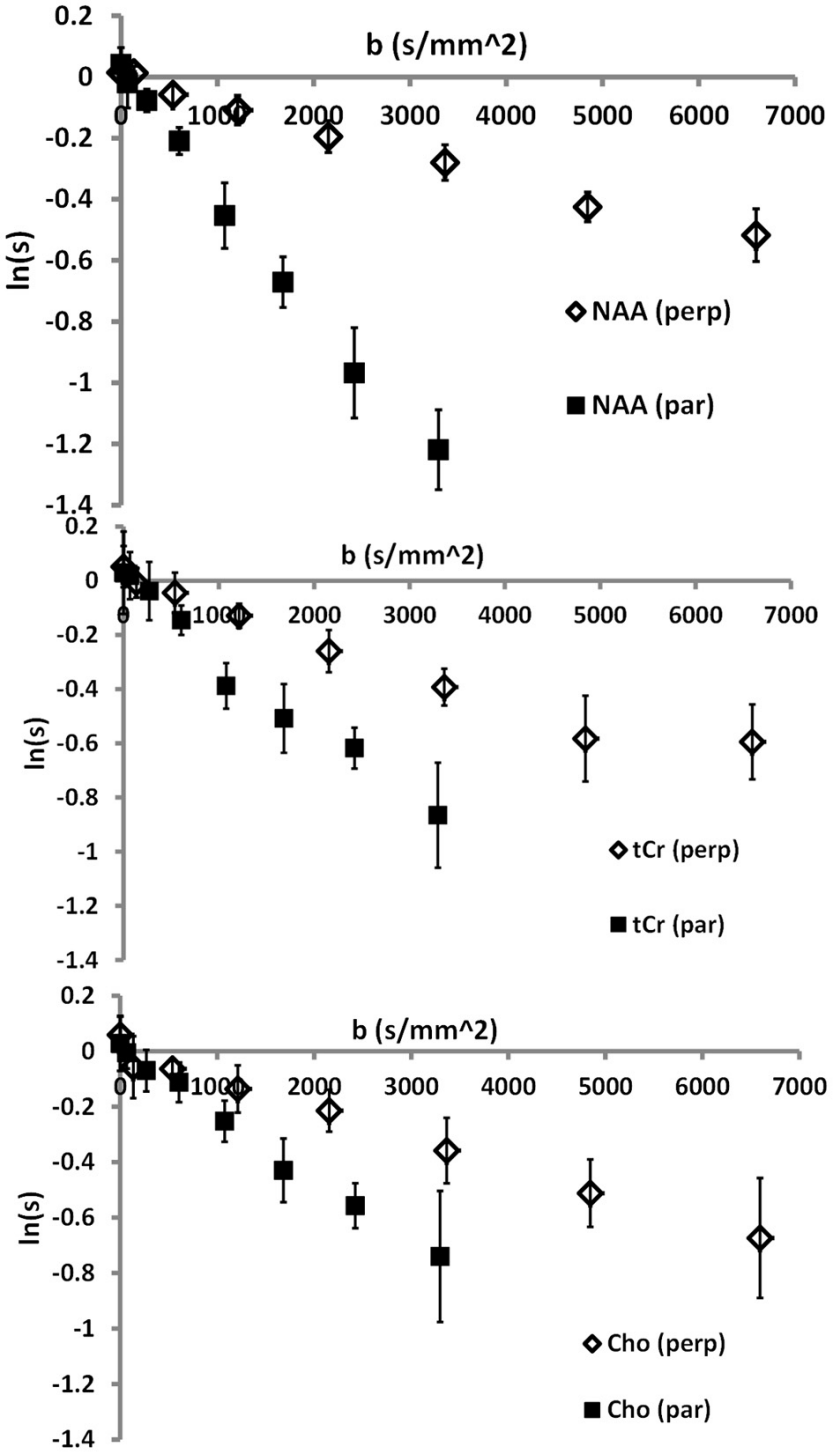
**Group-averaged data for the three main metabolites: top—tNAA; middle—tCr; bottom—tCho**.

Typical diffusion-weighted spectra are shown in Figure 4. The spectrum in the top panel was acquired without diffusion weighting. The one below was acquired with the largest *b*-value in the direction [0,1,1], which is mostly perpendicular to the callosal fibers. The bottom spectrum was acquired with *b* = 2422 s/mm^2^ in the [1,0,0] direction, which is roughly parallel to the callosal fibers. It is seen that the difference between the tNAA peak heights in the two diffusion-weighted spectra is significantly larger than the difference between the peak heights of the tCho and tCr. DWS data for the three metabolites: tNAA, tCho, and tCr, averaged over the six subjects scanned for tNAA and the six subjects scanned for tCho + tCr are shown in Figure 5. A qualitative estimate of the diffusion directionality, or anisotropy, for each of the three metabolites is provided by calculating *D*_[1,0,0]_/*D*_[0,1,1]_, the ratio between a single diffusion coefficient calculated for the set of measurements of diffusion parallel, and one calculated for the diffusion perpendicular, to the fibers. Table 1 summarizes these data. It can be seen that *D*_[1,0,0]_/*D*_[0,1,1]_ is the highest for tNAA (above 5), roughly twice the value for tCho and tCr. Interestingly, the values for *D*_[0,1,1]_ are rather similar for all three metabolites, *D*_[0,1,1]_ for tNAA being slightly lower than the other two, and substantial differences are found between *D*_[1,0,0]_ of tNAA and those of tCr and tCho.

**Table 1:** **Group-averaged DWS data (*n* = 6 for tNAA and *n* = 6 for tCr and tCho) for the three metabolites**.

	**ADC (mm^2^/s)**	***D*_⊥_(mm^2^)**	***D*_‖_(mm^2^/s)**	***D*_‖_/D_⊥_**	**D(model) (mm^2^/s)**	σ_ψ_(°)	***P*_glia_**
tNAA	0.24 ± 0.01	0.079 ± 0.003	0.41 ± 0.01	5.2 ± 0.3	0.48 ± 0.05	21.1 ± 2.6	–
tCr	0.18 ± 0.01	0.095 ± 0.004	0.26 ± 0.02	2.8 ± 0.4	0.37 ± 0.06	–	0.4 ± 0.1^*^
tCho	0.16 ± 0.02	0.093 ± 0.007	0.22 ± 0.02	2.4 ± 0.4	0.42 ± 0.08	–	0.5 ± 0.1^*^

* *p = 0.011, paired t-test*.

### Modeling of tNAA signal

**Figure 6:**
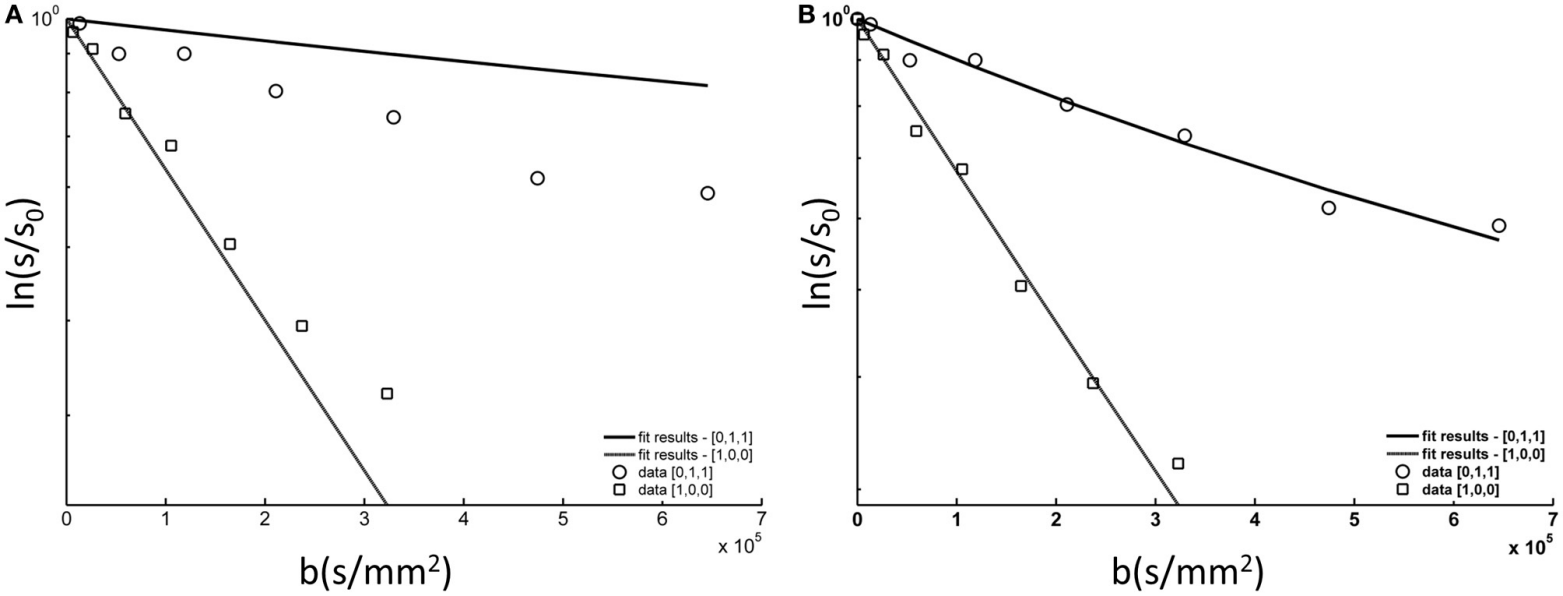
**tNAA DWS data from a single subject (symbols) fitted to the model described in the methods section and in step (A) in Figure 3. (A)** The data fitted to the model with one fitting variable, D(tNAA), and σ_ψ_ = 10.0°; **(B)** the data fitted to the model with two fitting variables, D(tNAA), and σ_ψ_. In this case D(tNAA) = 0.43 × 10^-3^ mm^2^/s and σ_ψ_ = 22.3°.

Figure 6 shows the DWS tNAA results from a single subject, to which the model described in the method section was fitted. The model fits the data well, well within the goodness-of-fit acceptance criterion [χ^2^_(14)_ < 6.3, *p* > 0.9 for all subjects]. The two fitting variables were σ_ψ_, the standard deviation of the axonal angular dispersion, and D(tNAA), the diffusion coefficient of tNAA in the cytoplasm. In order to exemplify the effect of σ_ψ_, a suboptimal fit is shown in the left panel, in which σ_ψ_ is about 50% of its optimal value. In that case, the diffusion effect is underestimated for *D*_[0,1,1]_ (mostly perpendicular to the fibers) and overestimated for *D*_[1,0,0]_ (mostly parallel to the fibers). The average results for D(tNAA) and σ_ψ_ for the six subjects are given in Table 1.

### Modeling of tCho and tCr signal

Choline soluble compounds are found in astrocytes and neuron/axons at a ratio of about 3:1, whereas Cr and PCr are ubiquitous in neural tissue, regardless of cell type (Choi et al., 2007). A comprehensive analysis of the diffusion-weighted tCho and tCr signals should thus consider the contribution of both glial and axonal compartments, as is explicitly specified in Equation 7. In order to fit the data to the model in Equation 7, the axonal angular dispersion σ_φ_ found for the tNAA was used as an input parameter for the modeling of the two remaining metabolite data, and the only fitting parameters were the glial fractions for the two metabolites, *P*_gl_(tCho) and *P*_gl_(tCr), and the cytoplasmic diffusion coefficients of tCho and tCr, D(tCho), and D(tCr), respectively. The cell body radius was not a set as a free variable, but was gradually incremented until a stable fit with a constant error was obtained. This occurred at *r*_gl_ = 9 μm, which is well within the range of astrocyte cell body radius (Kuehnel, 2003). The model fits the data reasonably well, with χ^2^_(14)_ < 11.7, *p* > 0.5 for all subjects. The group averages of the diffusion coefficient and the glial fractions for tCho and tCr are given in Table 1. The difference between *P*_gl_(tCr) and *P*_gl_(tCho) was statistically significant (*p* = 0.011, paired *t*-test).

## Discussion

The estimated *D*_‖_ and *D*_⊥_ for the three metabolites measured in this work qualitatively display the most salient difference in compartmentation between tNAA, which is predominantly present in neuronal and axonal cytoplasm, and tCho and tCr, which are not unique to neurons and are also found in glial cells, mostly in astrocytes. In this work, the DWS data for tCr and tCho were acquired separately from those for tNAA, introducing inter-individual differences as a possible confound, and it is thus desirable to devise a shortened acquisition protocol that allows to acquire these data from the same subject. The good reproducibility of the DWS results for each metabolite, as can be appreciated from the results shown in Figure 5, is encouraging, and indicates that inter-subject variability is relatively low. From the structural perspective, it is useful to consider the intra-axonal space as cylinders with an inner diameter that is mostly in the 1–5 μm range (Aboitiz et al., 1992), and astrocytes as represented by slightly oblong oval structures with average diameter of about 10–20 μm (Glenn et al., 1992; Kuehnel, 2003) with thin processes that radiate radially from the soma in a rather isotropic fashion (Verkhratsky and Butt, 2007). It has been established that tNAA is almost exclusively intracellular (Huang et al., 2000), and thus any contribution from “hindered” diffusion in the extracellular matrix can be neglected. The picture may be somewhat more complex for tCr and tCho, which both cross the blood brain barrier and are then cleared from the extracellular space through specialized transporters, although in normal conditions the levels of both extracellular tCr and tCho are low compared to intracellular levels (Klein et al., 1991; Loffelholz et al., 1993; Ohtsuki et al., 2002).

The strong structural anisotropy of axons is reflected by the significantly higher *D*_[1,0,0]_ value for tNAA than that for tCr and tCho. A strong incentive for an accurate estimation for D(tNAA) is the suggested link between axonal integrity and the diffusion coefficient along the axonal axis, or axial diffusivity as evaluated from the diffusion tensor (Song et al., 2003; Kim et al., 2006; Budde et al., 2007). The axial diffusivity is the first eigenvalue of the diffusion tensor, λ_1_, and represents the diffusion coefficient in the main direction of the axonal tract. In DTI experiments, where the spatial resolution is on the order of a few millimeters, λ_1_ is mostly defined by two major factors: the “effective” diffusion coefficient of water molecules in the intra- and extracellular space at the given diffusion time, and by the axonal organization, “fanning,” or microscopic misalignment. The former is tightly related to the tortuosity of both the cytoplasmic as well as the interstitial medium, and it is hypothesized that structural damage to intra-axonal structures such as neurofilaments and microtubules resulting from pathological processes may affect the intra-axonal component of λ_1_. Accurate measurement of D(tNAA) has the potential to provide an additional source of information on intra-axonal structural integrity in white matter tracts, while avoiding the confound of the extra-axonal contribution. The potential sensitivity of D(tNAA) to pathology has been shown in the case of MS, where the axial diffusivity of tNAA in the corpus callosum was significantly lower in a group of MS patients and correlated well with behavioral outcome (Wood et al., 2012). The specificity of DWS of tNAA is offset by the low sensitivity of the method, which dictates significantly larger volumes than the typical DTI voxel and longer measurement times. One of the main problems resulting from using a large VOI is the impact of macroscopic curvature of the axonal tracts on the estimation of D(tNAA). In this work we showed that in the relatively simple case of the corpus callosum, it is possible to incorporate in the model a synthetic angular distribution of axonal directions. A simple and robust way to provide a realistic estimate for the macroscopic curvature can be based on an independent DTI data set, where the set of main eigenvectors for the DTI voxels within the DWS volume can be used to generate a realistic distribution of angles between the tract directions and the diffusion-weighting gradients. These angular distributions are patently non-Gaussian, and are expected to be different for each diffusion-weighting direction. We do not present such distributions in this work as we have not acquired DTI data from the subjects on which the reported DWS measurements were performed, but preliminary estimates based on data collected so far for a current project yield values of σ_θ_[1,0,0] ≈ 15° and σ_θ_[0,1,1] ≈ 10°, where θ is the angle between the diffusion-weighting direction ([1,0,0] or [0,1,1]) and the main eigenvector of a DTI voxel within the DWS VOI. The quantitative incorporation of these angular distributions in the processing pipeline of DWS data of NAA is one of the future directions we propose to investigate. Additionally, the addition of a DTI experiment to the DWS protocol will provide tractographic data for the visualization of white matter tracts of interest that are not as recognizable as the corpus callosum, and help position the DWS VOI on such tracts.

The similar *D*_[1,0,0]_ and *D*_[0,1,1]_ values for tCho and tCr initially suggest that the compartmentation of these two metabolites in white matter is rather similar, and the differences can at least partly be explained by differences in molecular weight and in interactions with the surrounding medium. Upon more detailed analysis of the diffusion data for these two metabolites, a significant difference in their glial fraction is found, namely, the glial fraction of tCho for each subject is significantly higher than the glial fraction of tCr. This finding is supported by the literature (Choi et al., 2007) and provides an encouraging support for the validity of the suggested model. It should be noted that since the measurements shown here were performed using a *single diffusion time*, it is impossible to use the data to extract information about compartment size, and in the proposed analysis the compartment size was either provided from histological data, as in the case of the axonal diameter, or given a lower limit in which the data fit well with the model, as in the glial diameter. The estimate of the latter (*r*_gl_ = 9 μm) agrees well with histological data, but there is no doubt that measurements with a set of diffusion times would narrow down the estimates for D(tCr) and D(tCho), as well as those for the glial fraction for those metabolites. Moreover, we assumed here that the prevalent geometry of astrocytes is that of spherical compartments. This is a simplistic assumption that does not take into account astrocytic processes, which comprise a significant fraction of the astrocytic volume (Perge et al., 2009). The astrocytic processes are isotropically distributed and thus do not affect the directional anisotropy of the DWS measurements, but since diffusion in fibers significantly differs from diffusion in spheres in its dependence on diffusion time (Marchadour et al., 2012) it is expected that introducing astrocytic processes to the model will have an impact on both the estimate of glial size as well as on D(tCr) and D(tCho).

Diffusion properties of intracellular metabolites are not only indicative of cellular microstructure. In a recent study (Branzoli et al., 2013), DWS was applied to measure changes in ADC of metabolites in the primary visual cortex upon visual stimulation. In this work, the most salient result was that the ADC of tCr robustly increased with activation, and correlated well with the stimulation paradigm. Although this finding does not exclude the possibility of microscopic neuronal structural changes upon activation such as cell swelling (Le Bihan et al., 2006), the fact that the ADC of tNAA, a purely neuronal constituent, was significantly *less* correlated with the stimulus indicate a possible involvement of a metabolic process as responsible to the increase in ADC of tCr, rather than a structural one. The increase in SNR at ultra-high field facilitated the robust separate assessment of ADC of glutamate and glutamine in gray and white matter (Kan et al., 2012). It is hoped that measuring the diffusion properties of glutamate and glutamine will provide further useful insights into such processes as glutamate release and the transport of glutamine from astrocytes to neurons upon neuronal activation, and contribute to the understanding of the role of glutamate compartmentation in disorders such as schizophrenia (Moghaddam et al., 1997; Moghaddam and Javitt, 2012).

In this work we provided some insight into the vast potential of obtaining unique compartment-specific information from DWS, and an example of a scaffold for the analysis of such data. There are significant challenges associated with robust acquisition, processing and analysis of diffusion-weighted spectroscopy. Additionally, the lower sensitivity of DWS when compared to diffusion-based imaging methods, prevents it from being used for high-resolution *mapping* of microstructural features of tissue. Conversely, the unique intrinsic compartment-specificity of the metabolites probed with DWS gives DWS a potential role in tissue characterization that water-based diffusion methods cannot fulfill. The challenge ahead is how to combine, in a multi-modal manner, DWS data with more sensitive but less-specific high-resolution data from e.g., DTI, to provide stronger explanatory power to both. Early attempts at doing so aimed at better understanding the processes that affect tissue in stroke (Wick et al., 1995; van der Toorn et al., 1996), and recently such attempt was aimed at characterizing tissue damage in normal appearing white matter in MS (Wood et al., 2012). DWS will certainly gain from the advent of ultra-high field MRI for humans (7T and higher), as the sensitivity of localized spectroscopy at higher fields is increased, with very few detrimental effects. It is hoped that the increase in availability of ultra-high field MRI scanners will generate an increase in interest in this unique tool, and that DWS will find its place in the growing arsenal of methods used for the non-invasive investigation of tissue microstructure.

### Conflict of interest statement

The authors declare that the research was conducted in the absence of any commercial or financial relationships that could be construed as a potential conflict of interest.
